# Preparation of N-Doped Carbon Nanosheets from Sewage Sludge for Adsorption Studies of Cr(VI) from Aqueous Solution

**DOI:** 10.3390/nano9020265

**Published:** 2019-02-15

**Authors:** Yi Wang, Weinan Zhao, Wanlan Zheng, Shuang Chen, Jinsheng Zhao

**Affiliations:** 1 State Key Laboratory of Heavy Oil Processing, College of Chemical Engineering, China University of Petroleum (East China), Qingdao 266580, China; wangiiupc@163.com (Y.W.); zhaoweinan65@gmail.com (W.Z.); zhengyouxi-1@163.com (W.Z.); 2 Shandong Key Laboratory of Chemical Energy Storage and Novel Cell Technology, Liaocheng University, Liaocheng 252059, China

**Keywords:** sewage sludge, nitrogen-doped carbon nanosheets, Cr(VI) adsorption, recycled materials, DFT calculation

## Abstract

Porous activated carbon with specific morphology and structure are of particular importance for waste water treatment, especially for the adsorption of toxic hexavalent chromium Cr(VI). However, the scalable and cheap production of such absorbents still suffer a grand challenge. Herein, a new type of N-doped nanosheet was innovatively prepared from easily available and low-cost sewage sludge via a facile and recyclable KOH activation method. The N-doped porous carbon nanosheets (N-SAC) produced by introduction of KOH and dicyandiamide, which performed favourable features for metal ions adsorption (93.2% for Cr(VI)) due to its high specific surface area, tuneable pore size distributions and good hydrophilicity. Additionally, the capacity also remained high after two cycles of adsorption by thermal regeneration, with 90.8% removal rate. The DFT calculation also approved that the doping of N could optimize the Mulliken charges distribution and improve the HOMO energy and improve the adsorption ability of N-SAC. This original proposal may inspire new possibility of creating porous carbon absorbents in a recyclable method.

## 1. Introduction

Water pollution, arising from the discharge of sewage, is imposed as a serious threat to ecological balance in the environment [1,2]. The accumulation of organic micropollutants, highly toxic substances, such as heavy metals, PAHs or pesticides, were found in water and it became one of the biggest challenges to public health and ecosystems today. Thus, removing these contaminants more effective by using efficient and cheap materials is highly desired. 

Poisonous metal ions [3,4] is the most dangerous contaminant due to its high carcinogenicity and teratogenicity that cannot be decomposed. And Chromium, a typical heavy metals containment, which mainly existing in two state: Cr(III) and Cr(VI) in the natural environment. Cr(III) is recognized as a micro nutrient for human requirement at a low concentration, whereas Cr(VI), from industry effluents, only presents a poisonous performance after accumulating in human system which could have an adverse effect on people’s blood [5,6,7], bone and nerve transmission. Therefore, it is highly required to remove Cr(VI) from waste water. There are various advanced treatments to reduce the harmfulness of Cr(VI) discharge, such as chemical precipitation, bio flocculation, adsorption, membrane separation and so forth. But the most practical and effective approach is adsorption that based on absorbents, such as activated carbon. While activated carbon is expensive non-recyclable and needs proper condition of use, the further application and utilization of adsorption method is limited for waste water treatment. 

From a sustainable viewpoint, the seeking for renewable carbon source is of critical importance in bringing carbon-based absorbents into practical waste water treatment [8,9]. However, some useful strategies developed so far for improving the adsorption capacity from naturally abundant biomass, including cellulose, hemicelluloses and lignin which represents the most promising precursor for sustainable activated carbon production [10,11]. The high price and low utilization still cannot implement recycle and reuse [7]. On the other hand, the cheap biomass, such as chicken eggshell, prawn shells, soybeans, human hair, garlic skin and ant powder [12,13,14,15], have also been extensively investigated as raw materials for carbon materials. But it was constrained by its low adsorption efficiency and complicated synthesis process. 

Sewage sludge, as a kind of hard-to-dispose yet rich organic matters [16,17,18], has been used mainly in landfills or directly burned [19]. It will also cause great waste of resources and damage on the environment. Taking account of high contents of aromatic and high carbon yield in sewage sludge, it is necessary to utilize its available advantages to fabricate high-value-add carbon adsorbent towards waste water pollution.

Herein, we demonstrated a facile and recyclable method to synthesize nitrogen-doped porous carbon nanosheets (N-SAC) via sewage sludge through a mildly modified KOH activation process [20]. Under KOH activation process, the biomass can be converted into carbon material with large specific surface area and large porosity and the adding of dicyandiamide served as soft template to form the nanosheets morphology. Due to these structural and morphological merits, the N-SAC has achieved a high adsorption performance of Cr(VI) in a wide pH range (93.2%). And successful regeneration process also demonstrated the high utilization of N-SAC. Besides, the DFT calculation explored the mechanism of high performance of adsorption via Mulliken charges distribution, HOMO energy and adsorption energy. It is not only can fabricate carbon adsorbents by a facile and recyclable way but also provides a new possibility for synergistic treatment of sewage and sludge. 

## 2. Materials and Methods 

### 2.1. Materials Synthesis 

Sewage Sludge (SS) were obtained from a waste water plant in Dong Ying, northeast of China. KOH and dicyandiamide was bought from Aladdin Ltd. Distilled water was used throughout the experiments. All other chemicals were of analytical grade and used without any further purification process.

Preparation of sewage sludge carbon (SSC): The sewage sludge was derived from wastewater treatment plant. Firstly, the sewage sludge was washed by distilled water and ethanol to remove dust and other inorganic impurities and then dried at 60 °C for 24 h. After that, it was ground and screened from 40 to 100 mesh to filtrate the appropriate size that stored for further studies. The contents of moisture, volatile material, ash and fixed carbon of SS were determined according to national standard (GB/T 12496.3−1999) [21]. Then, amount of 5.0 g of the SS material was introduced into porcelain crucible, which was heated in tubular reheating furnace applying a temperature program of pre-carbonized at 550 °C for 1 h under nitrogen atmosphere which named sewage sludge carbon (SSC).

Preparation of SAC and N-SAC: The sludge-based activated carbon (SAC) and N-doped sludge-based activated carbon (N-SAC) were prepared from a step of balling with SSC and KOH and dicyandiamide at the mass ratio (1:3:0, 1:3:1). Then the mixtures were heated to 800 °C for 2 h at a heating rate of 5 °C min^−1 in N^_2_ atmosphere. Finally, the carbon materials were collected through repeated washing with the dilute HCl solution (2 M) and distilled water for 10 h and then dried at 105 °C overnight. After cooling down to room temperature, SAC and N-SAC were obtained.

Preparation of N-SAC’: The adsorbed N-SAC was centrifuged from solution and calcined at 800 °C for 2 h with a heating rate of 5 °C min^−1^ in N_2_ atmosphere to regenerate the adsorbent. And then the products were gathered by repeated washing with high concentration of potassium hydroxide and distilled water. After dried at 105 °C, N-SAC’ were obtained.

### 2.2. Adsorption Characterization 

For the adsorption studies, a stock solution of 100 mg·L^−1^ was prepared by dissolving 50 mg of K_2_Cr_2_O_7_ in 1000 mL of deionized water. The working solutions were prepared by diluting of the stock solution and the PH adjusted with HCl (0.1 mol·L^−1^) and NaOH (0.1 mol·L^−1^) solutions.

The pH effect was evaluated by 50.0 mL of Cr(VI) solution with initial concentration of 10 mg·L^−1^ and pH ranging from 2.0 to 8.0. Aliquots were placed in contact with 0.1g of N-SAC and shaken by 2 h in a shaker incubator at 25 °C. Then, Cr(VI) remaining concentrations were determined by a HASH water quality analyser (DR2800, U.S.A). The maximum amount adsorbed (q_m_) was calculated by Equation (1) [22]:

q_m_ = [(C_0_ − C_r_)/V]/m (1)

where C_0_ and C_r_ are initial and final concentrations of Cr(VI) (mg·L^−1^), respectively, V is the solution volume(L) and m is the mass of N-SAC(g).

The experiments of adsorption kinetics were performed from the mechanical stirring in time intervals ranging from 5 to 120 min, using a shaker incubator. Aliquots of 50.0 mL of Cr(VI) solutions with concentrations of 5.0, 10.0 and 15.0 mg·L^−1^ (pH = 6.0) were placed in contact with 0.1 g of N-SAC. After stirring, Cr(VI) remaining concentrations were determined. The amount of Cr(VI) adsorbed at time t (q_t_) [23] were calculated using Equation (2):

q_t_ = [(C_0_ − C_t_)/V]/m (2)

where C_0_ and C_t_ are initial and final concentrations of Cr(VI) (mg·L^−1^), respectively, V is the solution volume(L) and m is the mass of N-SAC(g).

Non-linear equations of kinetic models of pseudo-first-order and pseudo-second-order and isotherm models of Langmuir and Freundlich were fitted to the experimental data, to assess the dynamics and adsorption capacity. The model fits were evaluated from the normalized standard determination coefficients(R^2^). 

Internal diffusion model (IPD) was used to explain the adsorption mechanism and determine the rate control steps in the adsorption process. IPD model was calculated using Weber-Morris formula as follow Equation (3) [24]:

*Q*_t_ = *K*_id_ t^(1/2)^ + *k*_0_ (3)

where Q_t_ is the equilibrium adsorption capacity of Cr(VI) (mg·g^−1^), respectively, *K*_id_ is the internal diffusion rate constant, k_0_ is a constant.

The effect of temperature in the Cr(VI) adsorption onto N-SAC was evaluated from thermodynamic studies. Aliquots of 50.0 mL of Cr(VI) solution of 10.0 mg·L^−1^(pH = 6.0) were placed in contact with 0.1g of N-SAC, in polypropylene flasks and stirred for 6 h using a shaker incubator at temperature of 15, 25, 35, 45, 55 °C. After the equilibrium time, the remaining Cr(VI)concentrations were determined and the adsorbed maximum amount calculated from Equation (2). The thermodynamic parameters, such as Gibbs free energy(∆G^0^), enthalpy change(∆H^0^) and entropy change (∆S^0^) were calculated from the equations (Equations (4)–(6)).

K_d_ = q_m_/c_m_ (4)

(5) $$\ln K_{d}=\frac{\Delta S^{0}}{R}-\frac{\Delta H^{0}}{RT}$$

(6) $$\Delta G^{0}=\Delta H^{0}-T\Delta S^{0}$$

where K_d_ is the distribution coefficient, R=8.314 J·mol^−1^·K^−1^ is the constant, T(K) is the adsorption temperature.

### 2.3. Density Functional Theory (DFT)

DFT calculation: has been carried out to investigate the effect of N doped active sites of adsorbents on Cr(VI) removal. All the calculations have employed the Gaussian 09 program. The B3LYP/6-31G+ (d,p) level functional was used to calculate adsorption energies and were corrected by BSSE. The adsorption energy (*E*_ad_) of the adsorbate was defined as the equation:

*E*(ad) = *E* (structure + HCrO_4_^−^) − *E*(structure) − *E*(HCrO_4_^−^) + *E*(BSSE)

## 3. Results and Discussion

### 3.1. Proximate Analysis and Yield of Activated Carbon (SAC, N-SAC)

The contents of moisture, volatile material, ash and fixed carbon of sewage sludge were measured and obtained as 4.93%, 35.80%, 14.35% and 44.92%, respectively. According to results, the sewage sludge has sufficient amount of organic matter which represented by the percentage of fixed carbon content. In the meantime, the content of ash in this sludge could provide a possibility for preparing activated carbon during high temperature calcination and acquire excellent adsorption capacity.

**Scheme 1:**
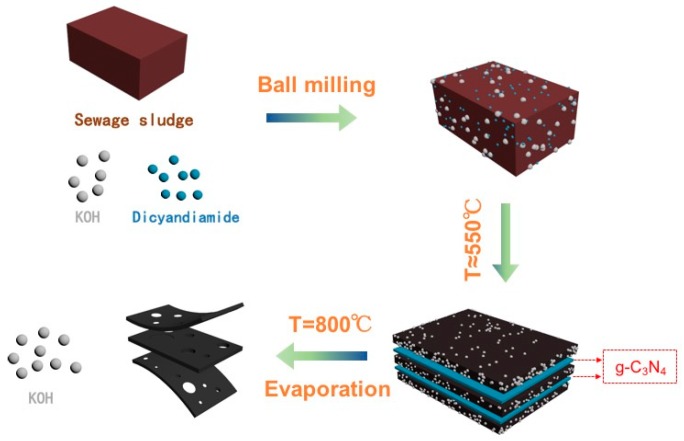
Illustration of the preparation procedure for the N-SAC nanosheets in recycled malt system.

The preparation process of N-SAC was shown in Scheme 1. Firstly, the sewage sludge carbon (SSC) was mixed with KOH and dicyandiamide. Then these mixtures were balled milling for 8 hours until blended completely and be calcined in N_2_ atmosphere. When the mixtures were heated approximately 550 °C, dicyandiamide was polymerized and generated g-C_3_N_4_ inside the SSC layer and the two-dimensional morphology of g-C_3_N_4_ carbon nanosheets could act as a soft template to guide and limit space of carbon mixture. Subsequently, when the annealing temperature was up to 800 °C, the decomposition of g-C_3_N_4_ could release gaseous nitrogen sources to dope into the carbon framework and produce the carbon nanosheets after all the g-C_3_N_4_ evaporation [11]. It is worth nothing that KOH could be easily recovered and reused to the next production of carbon nanosheets. The SAC were prepared according the same procedure except adding of dicyandiamide. Finally, the yields of SAC, N-SAC were as high as 54.3% and 56.1%, respectively and almost half weight loss could be assigned to the evaporation of volatile materials and ash from crude products. And the little higher yields of N-SAC compared with SAC due to the sufficient doped of nitrogen. 

### 3.2. Characterization

#### 3.2.1. Textural Properties

The textural properties of the SAC and N-SAC were evaluated from the N_2_ adsorption/desorption isotherms and Brunauer-Emmett-Teller (BET). 

**Figure 1:**
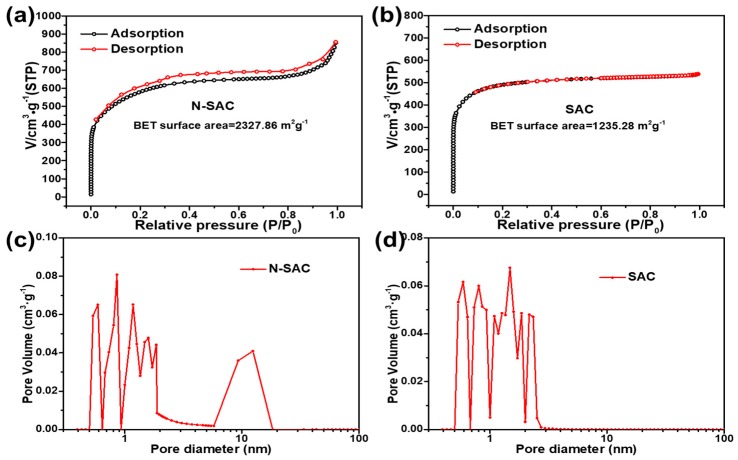
N_2_ adsorption-desorption isotherms and pore distribution calculated by DFT method for (**a**,**c**) N-SAC; (**b**,**d**) SAC.

The shape of the isotherm is related to type of solid porosity [25]. As shown in Figure 1a,b, the isotherms of SAC and N-SAC can be classified as type IV and hysteresis H4, presenting a rapid adsorption of N_2_ at low relative pressures and characteristic of microporous. The adsorption hysteresis loop appeared in medium relative pressure, which associated with capillary condensation that occurs in the mesoporous pores. Additionally, the narrow distribution of pore diameters for the SAC and N-SAC were calculated by DFT method in Figure 1c,d. As can be seen, the volumes of micropores (pore diameter < 2 nm) for SAC and N-SAC were similar, indicating that the chemical activation of KOH could react with active carbon framework during high temperature treatment, which gradually etched and generated the well-defined microporous framework. In addition, the volume of mesopores (pore diameter 2 to 50 nm) in N-SAC were much higher than SAC which could be attributed to the evaporation and decomposition of dicyandiamide during high temperature [21]. 

The textural properties of SAC and N-SAC were also displayed in Table 1. As can be observed, a significantly increase of D_ap_ value from 1.62 nm (SAC) to 5.50 nm (N-SAC), possibly driven by the reaction between CO_2_ and carbon, evaporating volatiles and increasing the pore sizes. Besides, SAC and N-SAC showed specific surface area (S_BET_) of 1235.2 m^2^ g^−1^, 2327.8 m^2^ g^−1^ respectively, which reached in this study are higher than other activated carbon (ACs) reported in literature [26,27] used sewage sludge as precursor. As a result, these merits of N-SAC could increase the contact area between adsorbent and waste water and expose more active sites for adsorbing Cr(VI) in solution. 

#### 3.2.2. Morphology Analysis

**Figure 2:**
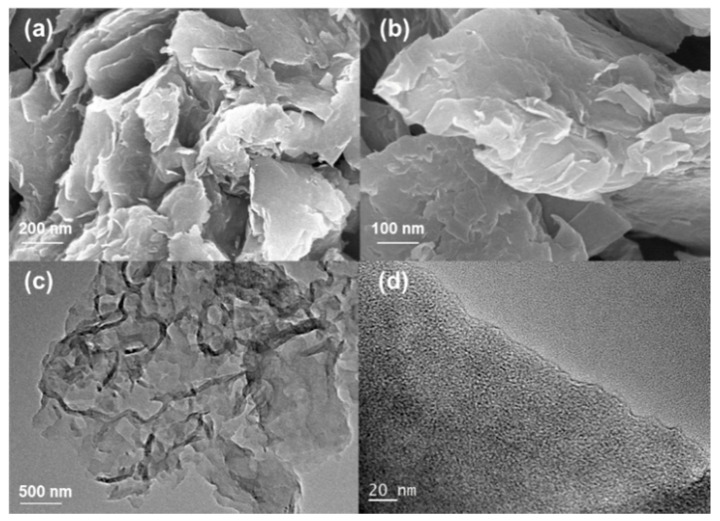
(**a**,**b**) SEM images of N-SAC; (**c**,**d**) TEM and HRTEM images of N-SAC.

The scanning electron micrograph (SEM) was carried out to evaluate the morphology of materials. As shown in Figure S1, it showed that raw materials, sewage sludge, have a compact structure without any cavities. After activated by KOH, SAC (Figure S2) has the loose structure, where the surface of the carbon is decorated with macropores. However, after the adding of dicyandiamide and calcination, N-SAC showed more open frameworks and two dimensional carbon nanosheets with wrinkled surface. Herein, the modification of N-SAC by dicyandiamide played an important role for guiding and expanding space during high temperature treatment. Firstly, the molecular of dicyandiamide was inserted into carbon matrix of SAC during the ball-milling process. Then, g-C_3_N_4_ was formed as the pyrolysis products of dicyandiamide at 550 °C which possessed sheet-like morphology and served as a template guiding the SAC carbon framework. Next, g-C_3_N_4_ were decomposition when the temperature is about 600 °C and reaction system has no residual g-C_3_N_4_ after it completely decomposed at 800 °C. These results were well in agreement with textural characteristics showed in Table 1, which surface area raised after modification, due to the activation and release of volatile compounds during carbonization process [12,28]. Moreover, the Transmission Electron Microscope (TEM) were test to verify the nanolattice structure. As shown in Figure 2c, the image exhibits the sheet-like structure which well match with SEM results. And in the high-resolution TEM (HR-TEM) picture, it could be observed that the surface of the nanosheets are full of micropores, which can secure large surface area and high porosity. Sewage sludge presents a dense surface without cavities, whereas SAC and N-SAC both show a rougher surface with cavities of various dimensions, indicating a well-developed porous structure especially on N-SAC. The pores on N-SAC surface provide suitable channels for the penetration of Cr(VI) ions into the carbon structure, allowing access to its mesopores and micropores, where they can interact with the surface functional groups. 

#### 3.2.3. FT-IR and XPS Analysis

**Figure 3:**
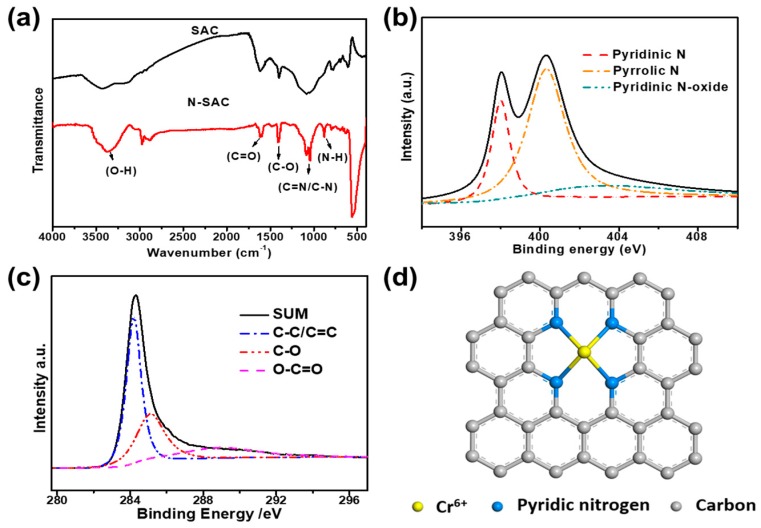
(**a**) Fourier transform infrared (FT-IR) spectra of N-SAC and SAC. (**b**,**c**) High resolution X-ray photoelectron spectroscopy (XPS) spectrum of N and C in N-SAC. (**d**) Possible hypothesis of chemical adsorption of Cr-N_4_ structure in N-SAC.

Fourier transform infrared (FT-IR) spectra were carried out to investigate the chemical bonds in SAC and N-SAC. As is shown in Figure 3a, SAC and N-SAC showed the characteristic of C=O and C-O stretching vibrations at 1650 cm^−1^ and 1425 cm^−1^ respectively [29]. However, the new peak at 966 cm^−1^ in N-SAC verified the formation of N-H which demonstrated the successful doped of nitrogen in carbon framework. In the meantime, the peak at 1020–1360 cm^−1^ can be caused by the stretching vibration and bending vibration of nitrogen-containing functional groups such as C=N and C-N [30] and the strong and broad peak at 3412 cm^−1^ in N-SAC can be assigned to the asymmetric stretching vibration of O-H bond of alcohols [5], phenols and a small number of amino groups adding with dicyandiamide.

Then, the X-ray photoelectron spectroscopy (XPS) were used to evaluate the composition and chemical bond in N-SAC carbon matrix. As shown in Figure 3b, the high-resolution N1s spectra demonstrated the successful doping of N into N-SAC and divided into three peaks, corresponding to pyridinic nitrogen (398.2 ± 0.3 eV), pyrrolic nitrogen (400.2 ± 0.3 eV) and pyridinic N-oxide (402.9 ± 0.2 eV) [17]. And in the C1s spectra, three major peaks with binding energy at 284.2 (± 0.3 eV), 285.1 (±0.3 eV) and 288.7 (± 0.3 eV) can be identified as C-C (C=C), C-O and O-C=O. The doping of N and O into N-SAC could effectively improve the electronic conductivity and stability and activate the sp2 carbon framework according to optimize the electron cloud density [27]. Furthermore, the doping of pyridinic-N could bond with Cr by coming into being the Cr-N_4_ structure (Figure 3d), which could also enhance the accessibility of adsorption of Cr in waste water performance greatly.

The water contact angle measurement was implemented to investigate the impaction of N-doping on wettability. As shown in Figure S3, the contact angle of 64.81° indicates a great hydrophilicity of N-SAC, comparing with SAC (85.29°), because the introduction of C-N bonds and O-H bonds can significantly facilitate the fully wetting of the active species. Furthermore, the increase of hydrophilicity could make N-SAC easy to contact Cr(VI) ions in liquor and enhance the adsorption ability.

### 3.3. Adsorption Studies of Cr(VI) on N-SAC 

In order to test the performance of sludge-based activated carbon for adsorption of Cr(VI) ions from aqueous solution, the activated carbon of SAC and N-SAC were used and investigate on different pH.

#### 3.3.1. Effect of PH

**Figure 4:**
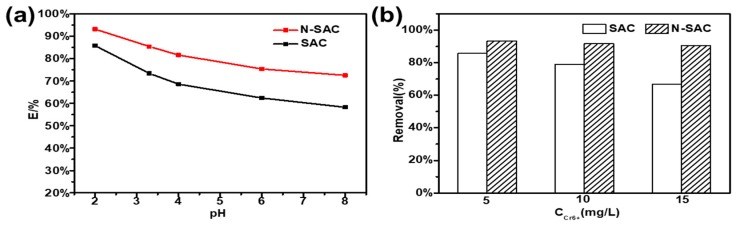
Kinetic model fitting at different concentrations: (**a**) Cr(VI) removal rates of SAC and N-SAC in different pH (2.0–8.0); (**b**) Cr(VI) removal rates of SAC and N-SAC in different concentration (5,10,15 mg L^−1^).

Figure 4a showed the adsorption capacity of SAC and N-SAC in terms of removal of Cr(VI) in different pH, ranging from 2.0 to 8.0. As can be seen, the pH of aqueous solution has a strong impact on the adsorption process as it can influence the charge of functional groups on the adsorbent surface and the chemical forms of chromium. 

In different pH value solutions, Cr(VI) can existed as chromic acid (H_2_CrO_4_), hydrogen chromate ion (HCrO^4-^) and chromate ion (CrO_4_^2−^) [31]. Under acidic conditions, the surface functional groups of the activated carbon combined with H^+^ in the solution to become protonated and positively charged, resulting that Cr (VI) reduced into Cr(III) via this reaction (Equations (4) and (5)). As shown in Figure 4a, the maximum removal rate (85.8%), can be attained at pH = 2 in SAC, while it fell to 58.2% at pH = 8.0. With the amount of OH^−^ increased, the decreased removal rate of Cr (VI) was the result of the surface electrostatic repulsion. The as-prepared N-SAC showed similar curve pattern, it could remove almost 93.2% of Cr (VI) in waste water on lower pH, which was much higher than that of SAC, because the nanosheets structure and mesopores could increase the contact area between adsorbent and Cr-contained solution and enhance the adsorbing capacity. Besides, the N-SAC suffered little impact from alkaline condition, Cr (VI) removal rate has decreased by 20.8% as the pH value varied from 2.0 to 8.0 in the solution, which was less than the decrease for SAC (25.6%) in the same process. It can be ascribed to the doping of N activated the sp2 carbon, which exposed more active sites for adsorption and improved the stability of carbon matrix in strong alkaline condition. Meanwhile, amino groups (-NH_2_) in N-SAC also played an important role in anion adsorption in high pH [32]. All these results indicated that N-doped modification on SAC was effective in the adsorption process of Cr(VI) by N-SAC.

#### 3.3.2. Comparison of Adsorption Performance

The operating condition also affected the adsorption capacity of adsorbents. Figure 4b showed the performance of SAC and N-SAC on the removal rate of Cr(VI) from different concentration aqueous at pH = 2.0. When the solution concentration of Cr(VI) increased from 5 mg·L^−1^ to 15 mg·L^−1^, the removal rate of Cr(VI) on SAC has a drop from 85.8% to 66.7%. However, N-SAC showed 93.2% of removal rate at 5 mg·L^−1^ and its removal efficiency was only decreased by 5.9% as the concentration of Cr(VI) was doubled, indicating the higher adsorption capacity of N-SAC than SAC at high concentrations of Cr(VI). It elucidated that the nitrogen modification brought N-SAC with stable and high adsorption capacity in different conditions, which can be overcame the limitation of using condition in traditional activated carbon.

#### 3.3.3. Adsorption Kinetics 

**Figure 5:**
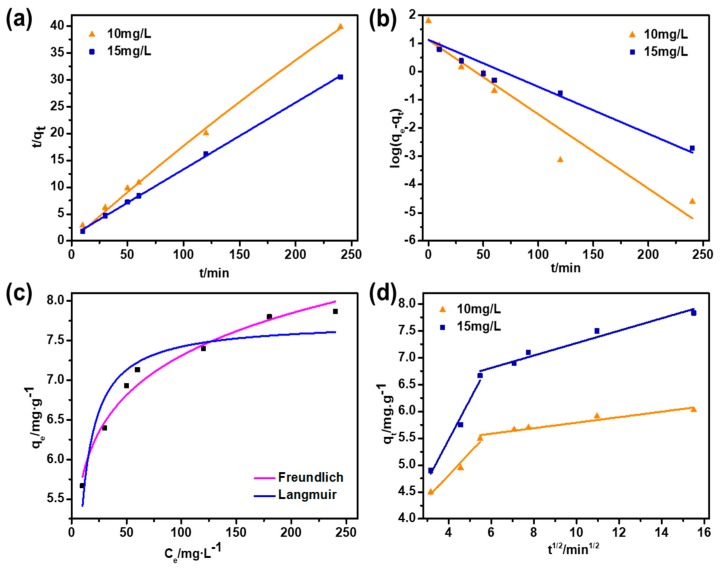
(**a**) Pseudo-first-order kinetic adsorption model fitting; (**b**) Pseudo-second-order kinetic adsorption model fitting; (**c**) Adsorption isotherm; (**d**) IPD fitting with N-SAC.

In order to investigate the adsorption dynamics of Cr(VI) on N-SAC, the kinetic models of pseudo-first order and pseudo-second order were fitted to experimental data and the linear fits are shown in Figure 5a,b [33,34]. According to the results (see Table 2), the calculated correlation coefficient value of pseudo-second-order (R^2^= 0.9984) is much higher than that of pseudo-first-order model (R^2^ = 0.9184), indicating that the pseudo-second-order model was suitable to describe the adsorption kinetic of Cr(VI). As the concentration of Cr(VI) increased, the rate constant (k) of C_Cr(VI)_ = mg·L^−1^ is higher than that of 15 mg·L^−1^ [31], indicating the decreased adsorption rate of Cr(VI) as the its initial concentration increased. The values of k2 were decreased from 0.0264 to 0.0186 mg.min^−1^ as the initial Cr (VI) was from 10 mg·L^−1^ to 15 mg·L^−1^, which was attributed to the lower competition for the adsorption surface sites at lower concentration and the active sites on the surface can adsorb Cr(VI) ions faster, so the pseudo-second-order kinetic rate constant is higher at lower Cr (VI) concentration. The result further confirms that N-SAC possesses a high adsorption rate for Cr(VI), which is mainly attributed to its unique hierarchical porous structure combined with a high specific surface area.

#### 3.3.4. Adsorption Isotherm

The adsorption isotherm, which is an important parameter for investigating the distribution of adsorbent on the surface of the adsorbent material and estimating its adsorption capacity. Thus, Langmuir and Freundlich models were fitted to experimental data and the results are shown in Figure 5c [35].

Table 3 shows the isothermal parameters of Freundlich and Langmuir models. According to results, the Freundlich model was fitted well to experimental data. The Freundlich model assumes that adsorption occurs over a heterogeneous surface in multilayer, assuming that adsorbent surface sites have a spectrum of different binding energies [30]. 

#### 3.3.5. Intraparticle Diffusion Model (IPD)

The IPD fitting curve was shown in Figure 5d and the relevant parameters are summarized in Table 4. It can be seen that the fitting curve can be divided into two sections at different initial concentrations [34], which represented the adsorption process of N-SAC on Cr(VI) was dual-stage. The first stage (Step I) was the macroporous diffusion adsorption of N-SAC, Cr(VI) ions diffused from solution to the adsorption sites on the surface of N-SAC macroporous surface. In step II, Cr(VI) ions diffused into the internal microporous channels of N-SAC and adsorbed on the surface of micropores. Among these two stage, the diffusion rate (K_id2_) of micropores was obviously slower than that of macrospores (K_id1_), indicating that the diffusion in micropores should be the rate-controlling step [36]. With the increase of initial concentration of Cr(VI) from 10 to 15 mg·L^−1^, the diffusion rates were increasing, which elucidated the diffusion driving force of Cr(VI) accelerated the adsorption process.

#### 3.3.6. Adsorption Thermodynamics

The thermodynamic parameters (Table 5) showed that ΔH^0^ was 79.46 kJ·mol^−1^, indicating that the adsorption process was endothermic. While ΔS^0^ was positive number that illustrated the disorder degree of the adsorbent surface increased during the adsorption process. Chemisorption and physical adsorption can be judged by ΔG^0^ [37]. When the adsorption was physical adsorption, −20 < ΔG^0^ < 0 kJ·mol^−1^, otherwise, −400 < ΔG^0^ < −80 kJ·mol^−1^ [38]. In this experiment, ΔG^0^ is in −9.75 to −22.14 kJ·mol^−1^, indicating that the adsorption process of Cr(VI) on N-SAC was mainly physical adsorption-a reversible process, under certain temperature and pressure conditions adsorption would reach equilibrium.

### 3.4. Cr (VI) Adsorption Mechanism

XPS was conducted to measure the elemental species to confirm the adsorption process of Cr(VI) on SAC and N-SAC. In the Cr(VI) solution, Cr(VI) can be expressed by a series of negative ions whose form conversions were correlated with the pH value, including HCr_2_O_7_^−^, HCrO_4_^−^, Cr_2_O_7_^2−^ and CrO_4_^2−^. Normally, HCrO_4_^−^ group was the predominant form in solution with pH < 6.5 [39]. As shown in Figure S4a, there were obviously N-SAC corresponding to C 1s, O 1s, N 1s, while the additional Cr 2p peak was found out after adsorption. The spectra of the Cr 2p showed two major peaks of Cr 2p on N-SAC, which were corresponded to Cr 2p_3/2_ and Cr 2p_1/2_, respectively [40]. After peak differential analysis, the Cr 2p spectrum can be divided into three peaks (Figure S4c), corresponding to Cr(III) located at 576.3 eV (Cr 2p_3/2_) and 586.9 eV (Cr 2p_1/2_) and Cr(VI) located at 577.8 eV (Cr 2p_3/2_) [41]. It indicated that a part of Cr(VI) is reduced to the low toxic Cr(III) during the adsorption on N-SAC and the residual of Cr(VI) is removed by physical adsorption. It indicated that Cr(VI) adsorption including two process: a part of Cr(VI) is reduced to the low toxic Cr(III) as Equations (7) and (8) [42] and the residual of Cr(VI) is removed by physical adsorption.

HCrO_4_^−^ + 7H^+^ + 3e^−^ → Cr^3+^ + 4H_2_O (7)

Cr_2_O_7_^2−^ + 14H^+^ + 6e^−^ → 2Cr^3+^ + 7H_2_O (8)

where the most of electron donors (e^−^) are from amino groups in N-SAC.

Besides, the N 1s peak has decreased after adsorption (Figure S4a), illustrating that nitrogen species of N-SAC were related to the Cr(VI) reduction. This result showed that the adsorption mechanism of Cr(VI) were multi step, including chemical reduction and physical adsorption. In comparison, XPS spectrum of Cr(VI) adsorption on SAC only showed peaks corresponding to C 1s, O 1s and a little amount of Cr 2p (Figure S4b). The Cr 2p peak on SAC was just resolved into two peak of Cr(VI), at 577.8 eV (Cr 2p_3/2_) and 587.1 eV (Cr 2p_1/2_) [43], elucidating the Cr(VI) on SAC was merely physical adsorption (Figure S4d). 

In all, reduction occurred along with the adsorption processes and the reducing agent was nitrogen atom groups on N-SAC. After reduction, the generating Cr(III) species were immobilized onto the adsorbent and residual Cr(VI) ions were adsorbed on the surface and pore of N-SAC, which has a higher removal rate of Cr(VI) than that of SAC. This results can also be proved in DFT calculation.

### 3.5. DFT Calculation

**Figure 6:**
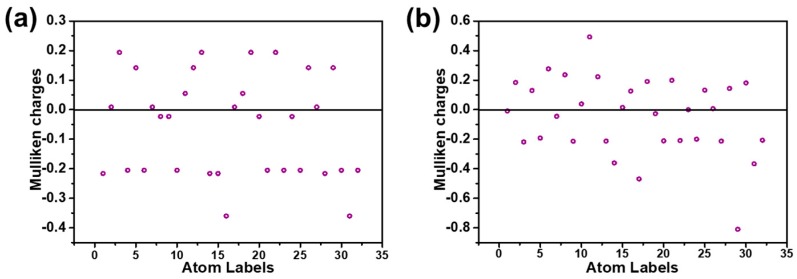
Mulliken charge distribution of Gra (**a**) and Gra-N (**b**) respectively.

To further understanding the primary effect of doping of N in carbon matrix, first-principles calculation was carried out to investigate the structure of simplified pure graphene model (Gra) and N-doped graphene model based on DFT methods and XPS results. Previous works demonstrated that atom with decreased charge density could serve as active sites for adsorbing. As shown in Figure 6, Gra-N model show lower Mulliken charge with −0.816, which value is much negative than pure Gra model (−0.381). These dates indicated that the doping of N could significantly decrease the Mulliken charge of adsorbent due to its high electronegativity, which may help materials to enhance its adsorption ability. Besides, the electronic cloud distribution at the highest occupied molecular orbital (HOMO) were also calculated on Figures S5 and S6. According to the frontier molecular orbital theory, the narrower of the gap between the HOMO of adsorbent the lowest unoccupied molecular orbital (LUMO) of adsorbent, the easier for the charge transfer process and the lower the adsorption energy. As shown in Figure 6 and Table 6, Gra-N presented higher HOMO energy and more negative adsorption energy than pure Gra, demonstrating the doping of N make it easier to capture HCrO4^−^ into carbon matrix during adsorption process. 

### 3.6. Regeneration Investigation

The study of thermal regeneration of the adsorbed carbon nanosheets (N-SAC) was carried out in one cycle(N-SAC`) and adsorption effect was shown in Figure S7. As it can be seen, the removal rate of Cr(VI) by N-SAC` still showed high adsorption capacity, at 90.8%, 88.6% and 86.19% in C_Cr(VI)_ = 5 mg·L^−1^, 10 mg·L^−1^ and 15 mg·L^−1^ respectively, which even higher than SAC in three different concentrations. It suggested that N-SAC` has high specific surface and enough active site after regeneration. As it shown in the Figure S8a, the phase impurity of N-SAC, including the crystalline Cr_2_O_3_ and C, which could be indexed based on crystalline Cr_2_O_3_ (JCPDS No. 85-0869, No. 06-0504) and Carbon (JCPDS No. 75-0444). After regeneration, Figure S8a showed that N-SAC’ sample only has a peak of C, suggesting that partial reduced Cr(III) in N-SAC was converted to metallic oxide Cr_2_O_3_ (Equations (9) and (10)) by carbon on high temperature and can be wiped out easily via caustic washing [44], which can be proved by XPS of N-SAC’(Figure S9). 

3^−^OH + Cr^3+^ → (^−^OH)₃Cr^3+^ (9)

2Cr(OH)_3_ → Cr_2_O_3_ + 3H_2_O (10)

The high adsorption efficiency of N-SAC` can be attributed to the reaction of Cr(VI) and Cr(III) adsorbed on the N-SAC during the thermal regeneration. N-SAC` can elute Cr_2_O_3_ easily and reuse on adsorption of Cr(VI) ion waste water [45,46,47]. 

## 4. Conclusions

In summary, a low-cost and convenient strategy has been developed to produce activated carbon nanosheets by using sewage sludge. Under the synergetic effect of KOH and dicyandiamide during the activation process, N-SAC showed good performance, such as high surface area, porous structure and high adsorption capacity of metal ions(Cr(VI). As a resultant, the adsorbent N-SAC showed its good adsorption property of Cr (VI) in waste water at 93.2% removal rate. Additionally, the recycled adsorbent still remained a high removal rate at 90.8% of Cr (VI) in solution after regeneration process. This facile route developed here may offer possibilities for mass production of biomass-derived carbon materials for waste water treatment and greatly promising for industrialization.

## Figures and Tables

**Table 1:** Textural information of SAC and N-SAC.

Sample	D_ap_ (nm)	S_BET_ (m^2^g^−1^)	S_mic_ (m^2^g^−1^)	V_t_ (cm^3^g^−1^)	V_mic_ (cm^3^g^−1^)	V_mic_/V_t_ (%)
N-SAC	5.50	2327.8	1736.1	1.35	0.97	71.8
SAC	1.62	1235.2	1126.2	0.62	0.59	95.1

D_ap_-average pore diameter; S_BET_-BET surface area; V_T_-total pore volume; V_mic_-micropore volume; V_mic_/V_t_-micropore percentage.

**Table 2:** Kinetic parameters of Cr(VI) adsorbed by N-SAC at different initial concentration.

C_0_ (mg·L^−1^)	q_e,exp_ (mg·g^−1^)	Pseudo-First Order			Pseudo-Second Order		
		q_e,cal1_ (mg·g^−1^)	k_1_ (min^−1^)	R^2^	q_e,cal1_ (mg·g^−1^)	k_2_ (mg·min^−1^)	R^2^
10	6.03	3.068	0.0263	0.9184	6.271	0.0264	0.9984
15	7.84	3.092	0.0167	0.08775	7.831	0.0186	0.9993

**Table 3:** Isothermal parameters of Cr(VI) adsorbed by N-SAC.

Langmuir	Freundlich
Q_m_ = 7.74 (mg·g^−1^)	k_F_ = 4.57
k_a_ = 0.23 (L·mg^−1^)	n_F_ = 9.81
R^2^ = 0.8773	R^2^ = 0.9710

**Table 4:** The IPD model parameters of Cr(VI) adsorption.

C_0,_ mg·L^−1^	C, mg·g^−1^	Step I		Step II	
		K_id1_	R^2^	K_id2_	R^2^
10	0.42	2.69	0.94	0.62	0.92
15	0.59	3.23	0.99	0.73	0.94

**Table 5:** The thermodynamic parameters of Cr(VI) adsorption.

ΔG^0^/kJ·mol^−1^					ΔH^0^ (kJ·mol^−1^)	ΔS^0^ (kJ·mol^−1^·K^−1^)
288K	298K	308K	318K	328K		
−9.75	−12.85	−15.95	−19.05	−22.14	79.46	0.3098

**Table 6:** HOMO Energy Level and adsorption energy on Different Models.

Adsorbents	HOMO Energy (eV)	Adsorption Energy (eV)
Gra	−5.57492	−0.1865
Gra-N	−3.39052	−0.5782

## Supplementary Materials

The following are available online at http://www.mdpi.com/2079-4991/9/2/265/s1. Figure S1: (a) and (b) The SEM images of raw materials of sewage sludge carbon (SS); Figure S2: (a) and (b) The SEM image of sludge-based activated carbon (SAC); Figure S3: Water contact angle measurement for (a) N-SAC and (b) SAC; Figure S4: (a), (b) X-ray photoelectron spectroscopy (XPS) of N-SAC and SAC before and after Cr6+ adsorption, (c), (d) High resolution XPS of Cr6+ after adsorption in N-SAC and SAC; Figure S5: (a) Atom label and (b) Mulliken charge density and (c) HOMO energy of carbon atoms in Gra model. Atom color code: yellow is carbon and blue is hydrogen; Figure S6: (a) Atom label and (b) Mulliken charge density and (c) HOMO energy of carbon atoms in Gra-N model. Atom color code: yellow is carbon and blue is hydrogen; Figure S7: The adsorption effect of Cr6+ removing from wastewater by SAC, N-SAC and N-SAC’, (a) removal rate, (b) color change of solution before and after adsorption; Figure S8: (a) XRD pattern of N-SAC after calcination, (b) XRD pattern of N-SAC’ after regeneration; Figure S9: X-ray photoelectron spectroscopy (XPS) of (a) N-SAC’, and high resolution XPS of (b) (c) (d) N, C and Cr respectively.
