# Supplementary material for: Preparation of N-Doped Carbon Nanosheets from Sewage Sludge for Adsorption Studies of Cr(VI) from Aqueous Solution
Source: Nanomaterials (Basel). 2019 Feb 15;9(2):265. doi: 10.3390/nano9020265 (PMC6410166; doi:10.3390/nano9020265)
Supplement: Supplementary file 1 [file nanomaterials-09-00265-s001.pdf]

Supporting Information

Preparation of N-doped carbon nanosheets from sewage sludge for adsorption studies of Cr(VI) from aqueous solution

Yi Wang [1,†], Weinan Zhao [1,†], Wanlan Zheng [1], Shuang Chen [1,*], Jinsheng Zhao[2,*]

1 State Key Laboratory of Heavy Oil Processing, College of Chemical Engineering, China University of Petroleum (East China), Qingdao 266580, P. R. China; wangiiupc@163.com (Y. Wang); zhaoweinan65@gmail.com (W.N. Zhao); zhengyouxi-1@163.com (W.L. Zheng).

2 Shandong Key Laboratory of Chemical Energy Storage and Novel Cell Technology, Liaocheng University, Liaocheng, 252059, P.R. China

* Correspondence: chsh1030@163.com(S. Chen); j.s.zhao@163.com (j.s.zhao)

† These authors contributed equally to this work.

Received: date; Accepted: date; Published: date

Received: date; Accepted: date; Published: date

This file includes supplementary Figures S1-S9.

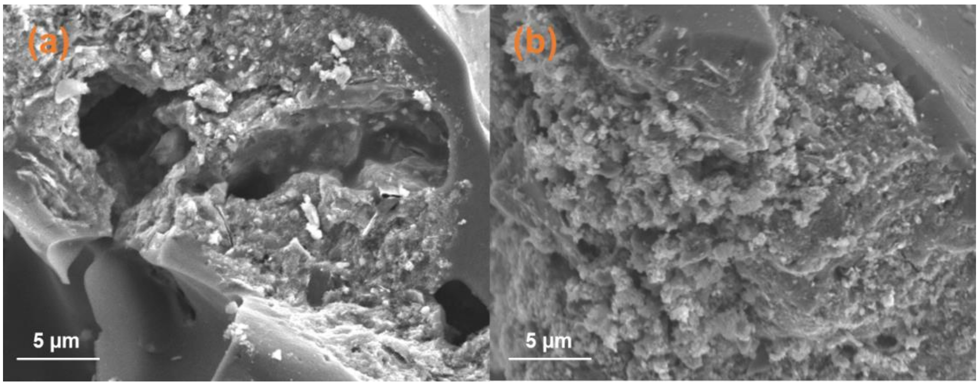

Figure S1. (a) and (b) The SEM images of raw materials of sewage sludge carbon (SS).

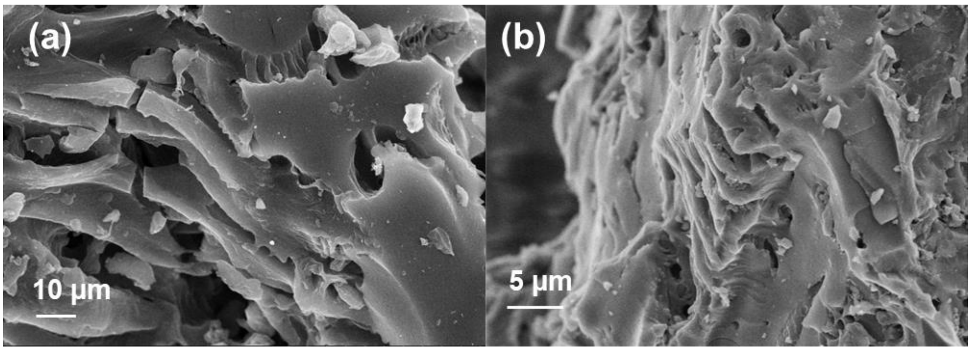

Figure S2. (a) and (b) The SEM image of sludge-based activated carbon (SAC).

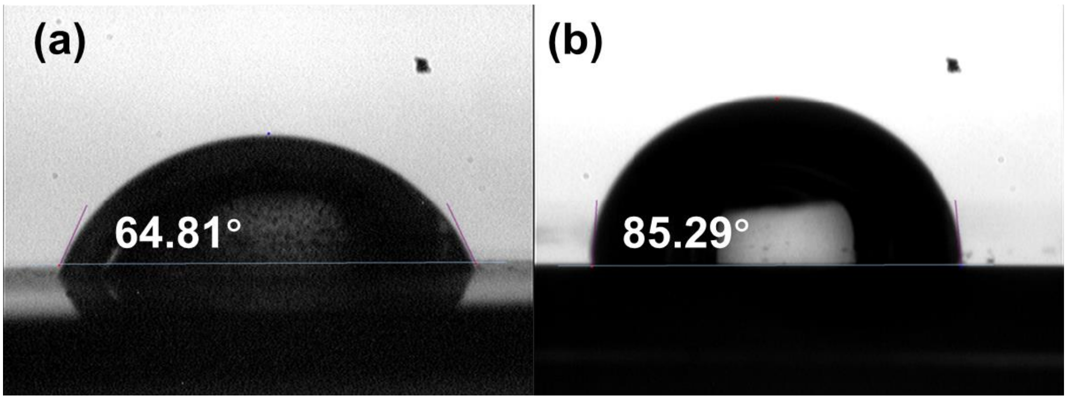

Figure S3. Water contact angle measurement for (a) N-SAC and (b) SAC.

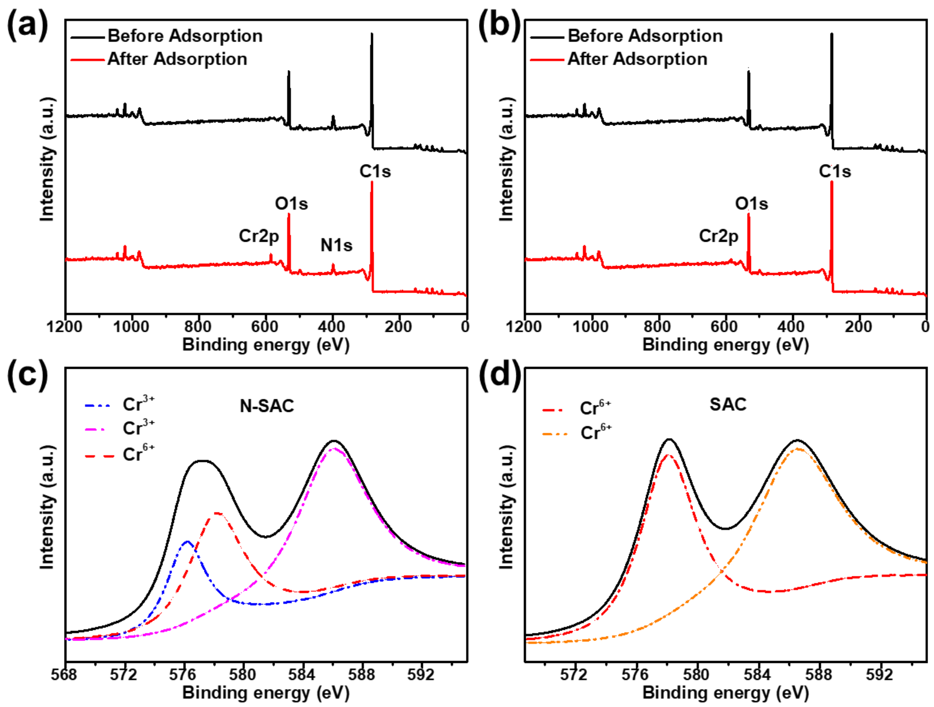

Figure S4. (a), (b) X-ray photoelectron spectroscopy (XPS) of N-SAC and SAC before and after Cr^{6+} adsorption, (c), (d) High resolution XPS of Cr^{6+} after adsorption in N-SAC and SAC, respectively.

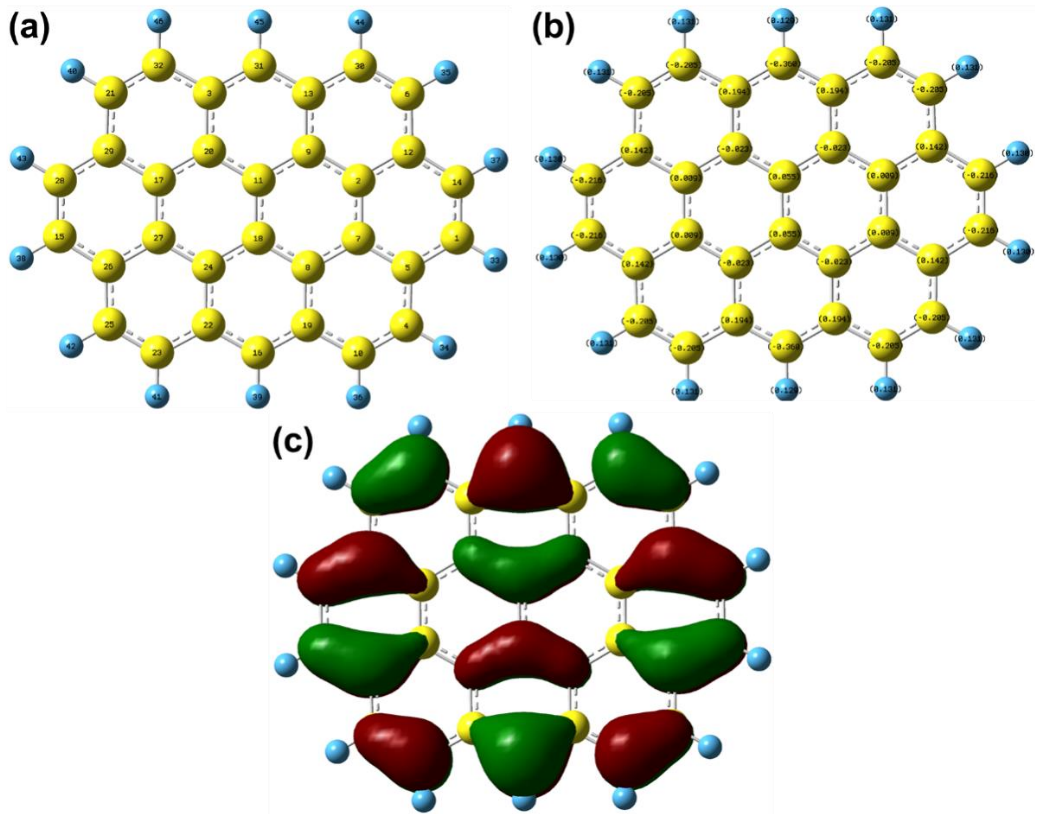

Figure S5. (a) Atom label and (b) Mulliken charge density and (c) HOMO energy of carbon atoms in Gra model. Atom color code: yellow is carbon and blue is hydrogen.

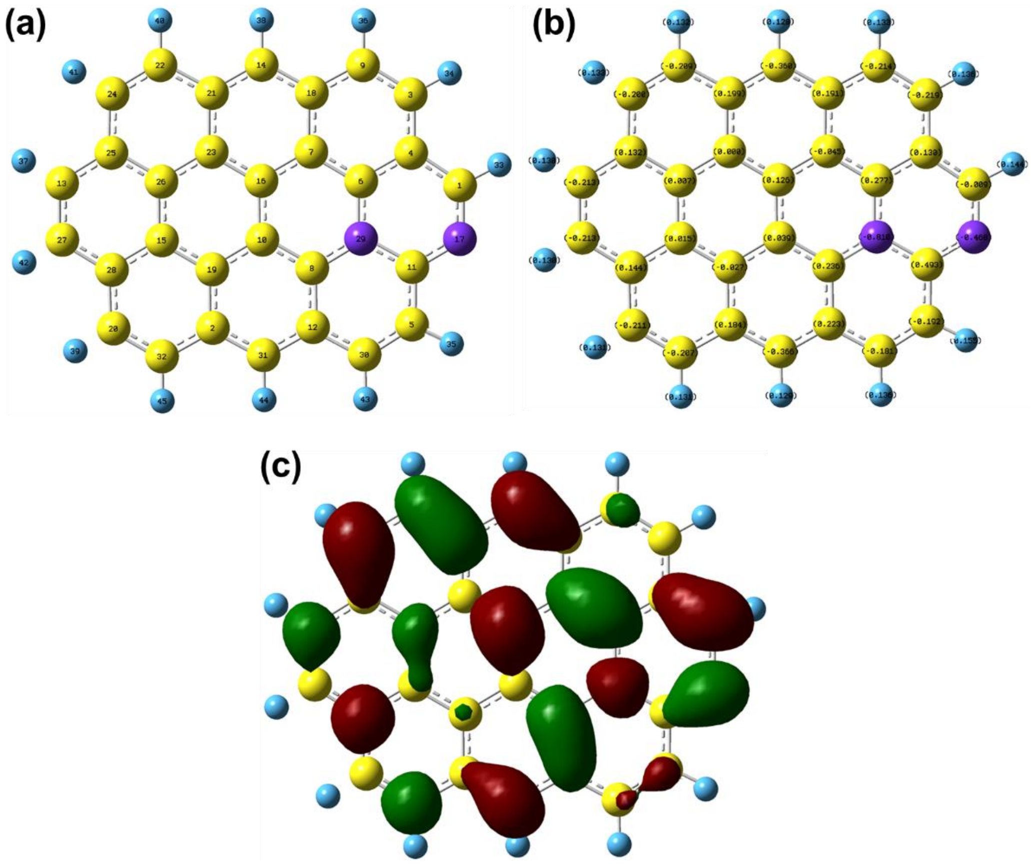

Figure S6. (a) Atom label and (b) Mulliken charge density and (c) HOMO energy of carbon atoms in Gra-N model. Atom color code: yellow is carbon and blue is hydrogen.

(a) (b)

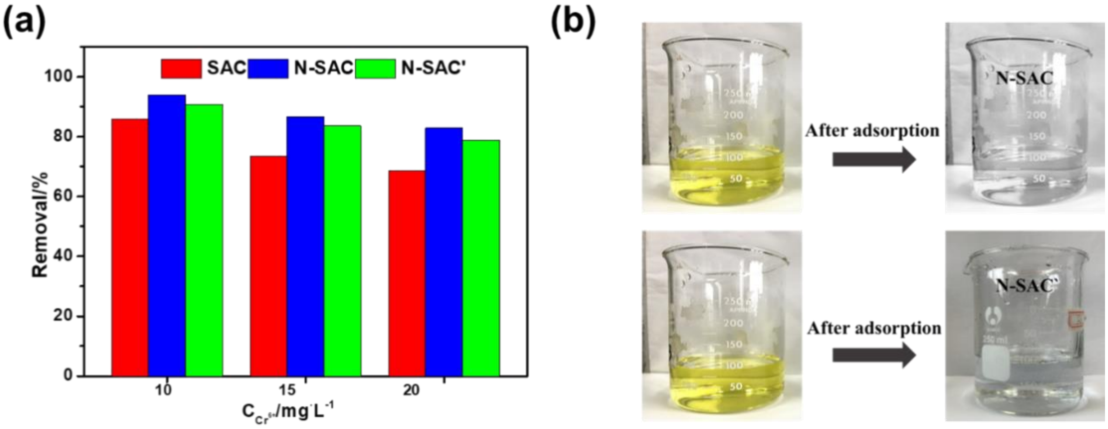

Figure S7. The adsorption effect of Cr^{6+} removing from wastewater by SAC, N-SAC and N-SAC', (a) removal rate, (b) Color change of solution before and after adsorption

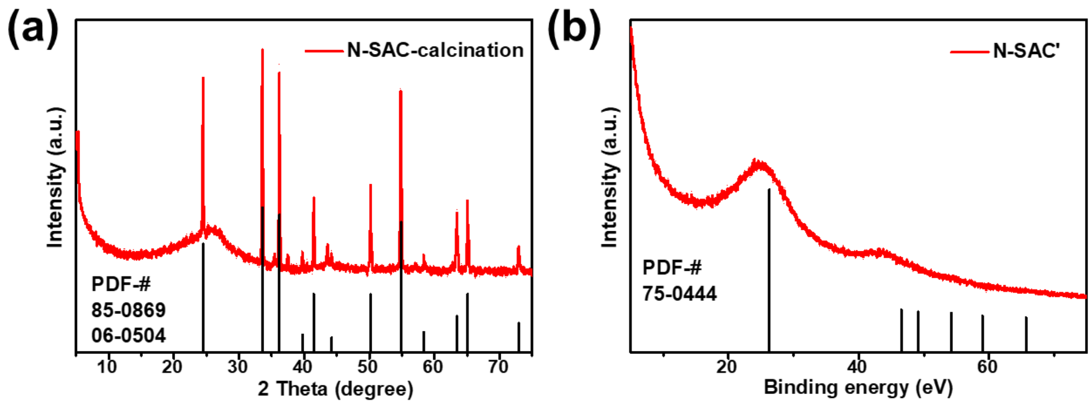

Figure S8. (a) XRD pattern of N-SAC after calcination, (b) XRD pattern of N-SAC' after regeneration.

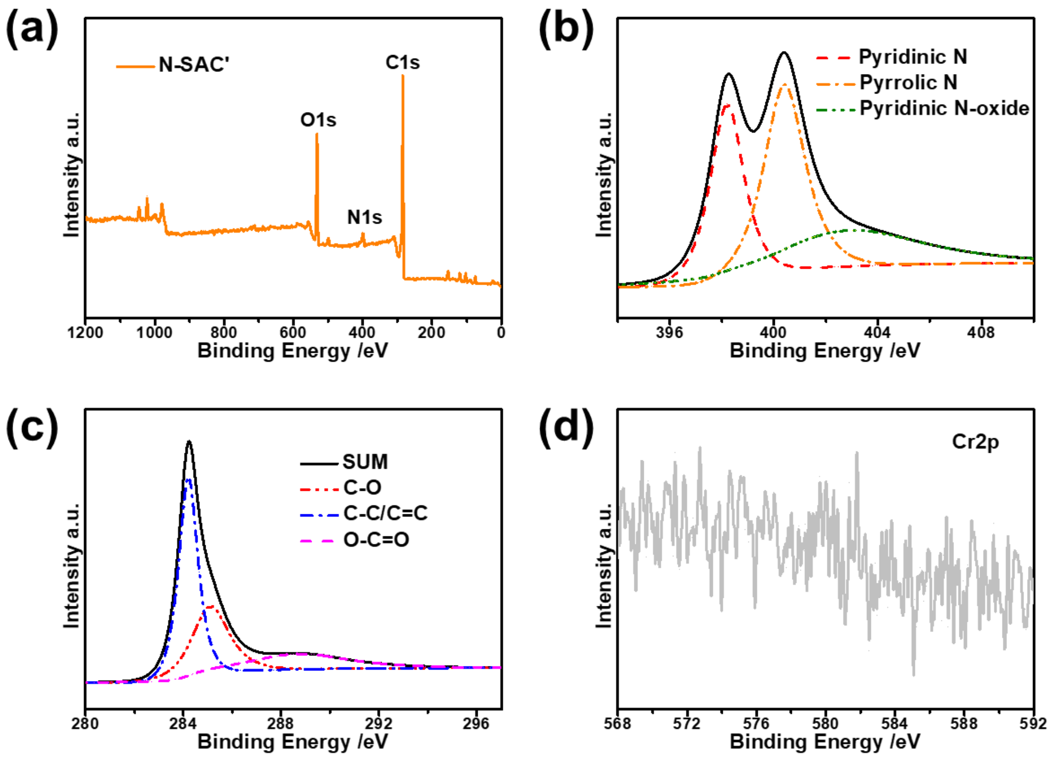

Figure S9. X-ray photoelectron spectroscopy (XPS) of (a) N-SAC', and high resolution XPS of (b) (c) (d) N, C and Cr respectively.
